# Sick Leave due to Stress and Subsequent Cancer Risk, a Swedish National Registry Study of 516,678 Cancer Cases

**DOI:** 10.1002/cam4.70888

**Published:** 2025-04-18

**Authors:** Jenny Hadrevi, Sai San Moon Lu, Lisbeth Slunga Järvholm, Richard Palmqvist, Tommy Olsson, Sophia Harlid, Bethany Van Guelpen

**Affiliations:** ^1^ Section of Sustainable Health, Department of Global Health and Epidemiology Umeå University Umeå Sweden; ^2^ Section of Oncology, Department of Diagnostics and Intervention Umeå University Umeå Sweden; ^3^ Section of Pathology, Department of Medical Biosciences Umeå University Umeå Sweden; ^4^ Section of Medicine, Department of Public Health and Clinical Medicine Umeå University Umeå Sweden; ^5^ Wallenberg Centre for Molecular Medicine Umeå University Umeå Sweden

**Keywords:** cancer, cervix cancer, exhaustion disorder, post‐traumatic stress disorder (PTSD), prostate cancer, sick leave, stress

## Abstract

**Background:**

This study examined whether sick leave due to severe stress (stress leave) and duration of leave are associated with future cancer risk.

**Methods:**

We conducted a matched case–control study using complete‐population data from Swedish national registers (2005 to 2018), including 516,678 primary cancer cases and 2,357,433 matched controls. Odds ratios (OR) were calculated by conditional logistic regression and adjusted for pre‐specified confounders.

**Results:**

Stress leave of any duration, reported to the Swedish Social Insurance Register, was associated with a slightly increased cancer risk, with the highest risk estimate for 1–30 versus 0 days (adjusted OR 1.05, 95% CI 1.02–1.09). In men, a clear exposure‐response trend was present. We observed increased risks of prostate cancer (adjusted OR for > 90 days: 1.10, 95% CI 1.01–1.20) and cervical cancer (adjusted OR for > 90 days: 1.11, 95% CI 1.05–1.17, including cancer in situ). In etiology‐based analyses, a positive association was found for smoking‐related cancers, and the risk relationship for non‐cervical HPV‐related cancers was similar to that for cervical cancer. Risk estimates were above one for several types of stress in relation to overall cancer risk, including an exposure‐response trend for acute stress reactions (p‐trend 4.0 × 10^−4^) but a null association for post‐traumatic stress disorder.

**Conclusions:**

Stress leave was associated with a modestly higher risk of cancer overall and prostate and cervical cancers specifically. Regardless of whether the link is biological or reflective of lifestyle mediators or for cervical cancer, lower participation in screening, these findings suggest a potential relevance of severe stress for cancer prevention.

## Graphical abstract

This population‐based registry study, including 516,678 primary cancer cases and 2,357,433 matched controls, investigated the relationship between sick leave due to severe stress, including the duration of leave and future cancer risk. Results show that sick leave due to severe stress was associated with a modestly higher risk of cancer overall and prostate and cervical cancers specifically, which may have relevance for cancer prevention.

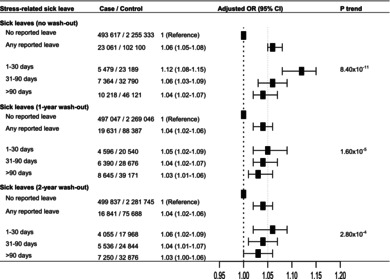

## 1 Introduction

Long‐term psychological stress can induce adverse health effects. This includes detrimental physiological effects [1, 2]. Notably, activation of the stress systems, including the hypothalamic–pituitary–adrenal (HPA)‐axis and the sympathetic nervous system, is implicated in several plausible mechanisms by which stress may contribute to carcinogenesis and tumor progression. These include altered endocrine and immune responses, inflammation, and key aspects of cancer initiation, such as DNA damage, repair, and epigenetic alterations [3, 4, 5, 6]. Chronic stress might also have an indirect but causal role in promoting cancer development through mediation by lifestyle‐related cancer risk factors. For example, tobacco smoking, alcohol consumption, and obesity are all overrepresented in individuals that have reported increased stress levels [7, 8].

Various types of psychological stress have been investigated in relation to cancer risk. Self‐reported work‐related stress generally shows null associations with cancer risk in prospective observational studies [9, 10]. For other stress‐related exposures, such as post‐traumatic stress disorder (PTSD), stressful life events, and adjustment disorders, some epidemiological evidence supports a positive association with the risk of cancer and specific types of cancer, though not consistently so [5, 11, 12, 13]. In a recent, very large, population‐based investigation using Swedish national registry data, patients with a stress diagnosis recorded in secondary (specialist) health care had a mildly elevated cancer risk, particularly for lifestyle‐related cancer types [11]. Taken together, findings to date raise the question of whether an increased cancer risk due to stress might become apparent at higher intensity and/or longer duration of stress exposure.

The aim of the present Swedish nation‐wide study was to examine exposure to severe stress, measured as sick leave and duration of leave registered in the Swedish Social Insurance Register, due to reactions to severe stress and adjustment disorders, in relation to subsequent risk of cancer and specific types of cancer.

## 2 Methods

### 2.1 Study Setting

**FIGURE 1:**
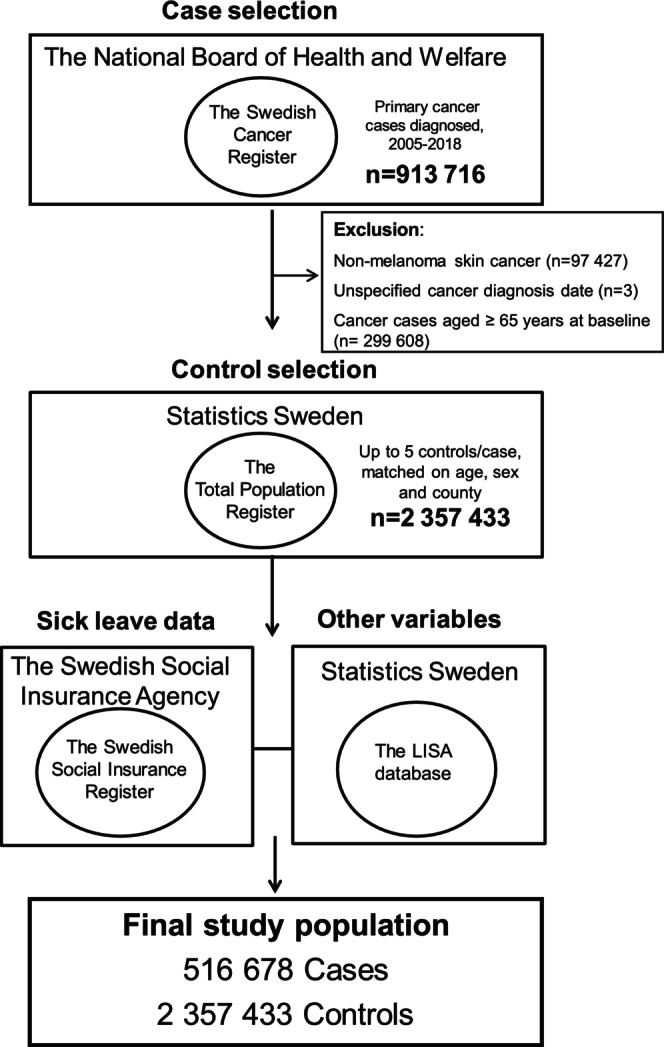
Flow chart of case and control selection. LISA = the Longitudinal Integration Database for Health Insurance and Labor Market Studies (LISA by Swedish acronym).

This was a matched case–control study nested within the Swedish population using nation‐wide register data from the period January 1, 2005, to December 31, 2018. Multi‐register linkage for case identification, selection of controls, and data acquisition were feasible through the use of Swedish unique personal identity numbers [14]. In short, primary cancer cases were identified using the Swedish Cancer Register, and controls were matched using the Total Population Register (RTB by Swedish acronym). The integrity of the patients was preserved by personal data being blinded by Statistics Sweden (SCB) upon data acquisition from registries. Stress‐related sick leave data were retrieved from the Swedish Social Insurance Register, and other covariate data were collected from the Longitudinal Integration Database for Health Insurance and Labor Market Studies (Longitudinell Integrationsdatabas för Sjukförsäkrings‐ och Arbetsmarknadsstudier), from 2005 for all variables (Figure 1 and Figure S1).

### 2.2 Selection of Cancer Cases and Controls

All primary cancer cases (except non‐melanoma skin cancer) diagnosed in Swedish residents aged 18 years or more and occurring between January 1, 2005, and December 31, 2018, were selected from the Swedish Cancer Register, which was founded in 1958 and covers the entire population of Sweden. All new cancer diagnoses are reported to the register, resulting in the registration of approximately 60,000 new cases every year. Reporting is obligatory by law, and completeness is around 96% [15]. The register contains individual patient data, comprehensive medical data such as ICD code, diagnosis date, and date of death.

Cases were categorized into 24 diagnosis groups (Table S1) based on ICD‐10 codes reported in the Swedish Cancer Register. We applied an arbitrary minimum threshold of 2000 cases in each diagnosis group in order to ensure a reasonable number of observations in the exposed groups. Moreover, cases were categorized into four groups based on etiology related to tobacco smoking [16], alcohol [17], obesity [18], gastric hyperacidity [19] and, in a post hoc analysis, human papilloma virus (HPV) [20] (Table S2).

For each case, up to five controls were randomly selected from the Total Population Register using incidence density sampling [21, 22]. Controls had to be born the same year, be of the same sex, and be living in the same county as their index case at the time of the cancer diagnosis of the case. Each control could only be included as a control for one case. Controls who were diagnosed with cancer (other than non‐melanoma skin cancer) after the cancer diagnosis of their index case were also included as cases, as of praxis. A total of 97,427 non‐melanoma skin cancer cases and 3 cases with incomplete information on cancer diagnosis date were excluded. We also excluded 299,608 cases who were of retirement age (> 65 years) at baseline, the year 2005, since the sick‐leave data is exclusively for the working‐age population. The final dataset comprised 516,678 cases and 2,357,433 matched controls (Figure 1). The mean follow‐up time was 7.5 years from the start of the study to cancer diagnosis.

### 2.3 Exposure Variables and Covariates

The study population was linked to the Swedish Social Insurance Register to retrieve information on sick leave due to reactions to severe stress, hereafter referred to as stress leave. The Swedish Social Insurance Register is administered by the Swedish Social Insurance Agency (Försäkringskassan in Swedish), a government agency responsible for compiling and publishing official statistics regarding social insurance for all Swedish residents [23]. Since 1994, the register includes sick leave and reimbursement information for “mental disorder” diagnosis groups (F00–F99), in addition to somatic diagnoses according to the Swedish version of ICD‐10. We included data from 2005 to ensure reasonably homogeneous reporting of the primary exposure diagnosis.

Stress leave data were collected using the diagnosis code F43, reaction to severe stress and adjustment disorders, and its subclassifications: acute stress reaction, F43.0; post‐traumatic stress disorder (PTSD), F43.1; adjustment disorder, F43.2 (encompassing reactions to major, life‐changing circumstances, such as the death of a child or divorce etc.); Stress‐induced exhaustion syndrome, F43.8A; Specified reactions to severe stress other than exhaustion syndrome, F43.8 W; and Unspecified reactions to severe stress, F43.9. In Sweden, the diagnosis of stress‐induced exhaustion disorder (F43.8A) was defined by the National Board of Health and Welfare in 2003 and has been used in clinical practice since 2005. This diagnosis is close to the burnout concept, and although it does not differentiate between types of stress causing the disorder, it is commonly due to work‐related stress [24]. For analyses using specific stress diagnosis codes within F43, we excluded exposures prior to 2010, due to substantial shifts in code use among the subclassifications within F43 during the period 2005–2010 (Figure S1). For analyses of overall stress leave (all F43), the full exposure period from 2005 was used.

The total numbers of reported days of stress leave reimbursed by the Swedish Social Insurance Agency during the study period and occurring prior to the cancer diagnosis of cases (and the corresponding date for their matched controls) were divided into four categories: none, 1–30 days, 31–90 days, and > 90 days. It should be noted that in Sweden, the first 2 weeks of sick leave are compensated by the employer and are, therefore, not included in the data from the Swedish Social Insurance Register.

Based on plausible roles as potential confounders of an association between stress leave and cancer risk and availabilities of variables available in national registries, we included an a priori selection of covariates in multivariable analyses: level of education (primary school up to 9 years, secondary school, postsecondary school, unknown), country of birth (Sweden, other Nordic countries, non‐Nordic European countries, non‐European countries, unknown), marital status (married/registered partner, unmarried, divorced/separated, widower/widow) and family disposable income (1st–4th quartiles). These data were collected from the LISA database (Longitudinell Integrationsdatabas för Sjukförsäkrings‐ och Arbetsmarknadsstudier). The LISA database, started in 1990, includes socioeconomic information for all residents of Sweden from 16 years of age, registered on December 31 each year. Inclusion in the register is compulsory, and the quality of the database is generally high [25].

### 2.4 Statistical Analysis

Multivariable conditional logistic regression, adjusting for the covariates listed above, was used to investigate associations between stress leave and subsequent cancer risk, reported as odds ratios (ORs) with 95% confidence intervals (CIs). The reference category was no reported stress leave during the study period, and risk estimates were calculated for the categories 1–30, 31–90, and > 90 days of reported stress leave. Trend tests were conducted by entering the exposure categories as an ordinal continuous variable coded 0–3 for no stress leave to > 90 days. The primary analyses were pre‐diagnostic stress leave in relation to overall cancer risk (tested with and without 1‐ and 2‐year wash‐out periods prior to cancer diagnosis) as well as the risk of specific types of cancer.

Prespecified secondary analyses included stratification by sex, analyses based on specific psychological stress diagnoses within the F43 diagnosis code, and analyses for cancer diagnosis groups based on etiology (cancer related to tobacco, alcohol, obesity, HPV and gastric hyperacidity).

All statistical tests were two‐sided and were performed using Stata/MP 16.1 (Stata Corp., College Station, TX, USA). *p*‐values < 0.05 were considered statistically significant except in the subgroup analyses of specific types of cancer, for which a Bonferroni‐corrected significance level of 0.002 was applied (alpha value divided by the 24 tumor types analyzed).

### 2.5 Ethical Approval

The study was approved by the Regional Ethical Review Board in Umeå, Sweden (Dnr: 2017‐194‐31 M and amendment 2019‐05046) and was conducted in accordance with the Declaration of Helsinki.

## 3 Results

The 516,678 cancer cases and 2,357,433 controls in the final study population demonstrated generally similar distributions with respect to birth country, education, marital status, and disposable family income, as well as for the matching variables sex, age, and county of residence (Table 1). Cancer was more common in women compared to men (57.5% versus 42.5%), and the mean age at cancer diagnosis was 55 years, reflecting the working‐age population. Nearly 30% of the cases were diagnosed with cancer prior to age 50 years. The mean follow‐up time was 7.5 years from baseline (start of the exposure period in 2005) to the cancer diagnosis of cases (and corresponding date for matched controls).

**TABLE 1:** Study characteristics.

Study characteristics	Cases (*n* = 516,678)	Controls (*n* = 2,357,433)
Sex, *n* (%)		
Men	219,594 (42.5)	955,170 (40.5)
Women	297,084 (57.5)	1,402,263 (59.5)
Continent of birth, *n* (%)		
Sweden	444,859 (86.1)	1,939,795 (82.3)
Other Nordic countries	20,925 (4.0)	88,072 (3.7)
Non‐Nordic European countries	27,058 (5.2)	150,031 (6.4)
Non‐European countries	23,824 (4.6)	179,371 (7.6)
Unknown	12 (< 0.1)	164 (< 0.1)
County, *n* (%)		
Region Stockholm	109,749 (21.2)	497,667 (21.1)
Region Skåne	70,636 (13.7)	317,255 (13.5)
Region Västra Götaland	89,270 (17.3)	404,498 (17.2)
Other regions	247,023 (47.8)	1,138,013 (48.3)
Education level, *n* (%)		
Primary school up to 9 years	126,253 (24.4)	554,338 (23.5)
Secondary school	226,721 (43.9)	1,008,437 (42.8)
Postsecondary school	152,927 (29.6)	709,968 (30.1)
Unknown	10,777 (2.1)	84,690 (3.6)
Marital status, *n* (%)		
Married/registered partner	257,948 (49.9)	1,184,898 (50.3)
Unmarried	167,500 (32.4)	786,282 (33.4)
Divorced/separated	80,089 (15.5)	338,661 (14.4)
Widower/widow	11,141 (2.2)	47,592 (2.0)
Disposable family income, *n* (%)		
1st quartile (lowest)	96,110 (18.6)	462,534 (19.6)
2nd quartile	111,352 (21.6)	493,636 (20.9)
3rd quartile	143,647 (27.8)	650,207 (27.6)
4th quartile	165,569 (32.0)	751,056 (31.9)
Age at baseline (2005), mean (SD)	48 (14.2)	47 (14.2)
Age at diagnosis, mean (SD)	55 (14.1)	—
Age at diagnosis, *n* (%)		
18–49 years	153,270 (29.7)	—
≥ 50 year (range 50–77 years)	363,408 (70.3)	—
Follow‐up time^a^, year		
Mean (SD)	7.5 (3.9)	—
Median (IQR)	8 (4–11)	—

Abbreviations: IQR = interquartile range, *n* = number, SD = standard deviation.

^a^ Follow‐up time from baseline (start of exposure period, 2005) to cancer diagnosis.

**FIGURE 2:**
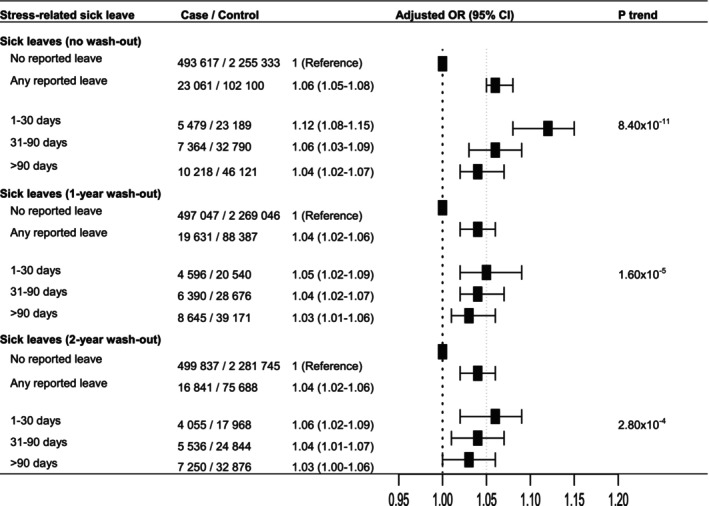
Stress leave and subsequent risk of cancer. Stress leave is defined as registered sick leave due to psychological stress with diagnosis codes (ICD‐10, International Classification of Diseases, 10th Revision) of F43, reaction to severe stress and adjustment disorders. In Sweden, the first 2 weeks of sick leave are compensated by the employer and are therefore not included in the national registry data used here. Washout represents excluding stress leave during the 1 or 2 years prior to cancer diagnosis to account for reverse causation. Conditional logistic regression, conditioned on matching factors (sex, age at diagnosis and county of residence) and adjusting for socioeconomic factors (level of education, country of birth, marital status and family disposable income), was used to calculate odds ratio (OR) with 95% confidence interval (CI). P‐trend was calculated by including the categories of stress‐leave duration as an ordinal continuous variable coded 0–3 for no stress leave to > 90 days in the multivariable analysis.

In the full dataset, stress leave of any duration, reported to the Swedish Social Insurance Register, was associated with a modestly increased subsequent risk of cancer (OR 1.06, 95% CI 1.05–1.08), with the highest risk observed for 1–30 versus 0 days reported during follow‐up (adjusted OR 1.12, 95% CI 1.08–1.15) (Figure 2). Excluding washout periods for stress leave during the 1 or 2 years prior to cancer diagnosis, to address potential reverse causality, attenuated the OR for the category 1–30 days, yielding consistent ORs of approximately 1.05 across durations of reported stress leave. A 1‐year washout period was therefore applied to all downstream analyses. The association between reported stress leave and cancer risk was present in both men and women (OR 1.05, 95% CI 1.01–1.09, and OR 1.03, 95% CI 1.01–1.05), respectively (Figure S2) whereas a clear exposure‐response trend was observed in men (p‐trend 0.005). For women, the ORs for all stress leave durations were above one but not linearly ordered by increasing duration of stress leave, despite the p‐trend of 0.006.

Risk estimates for stress leave due to specific types of stress reactions in relation to overall cancer risk are presented in Table 2. Odds ratios for any versus no reported stress leave were generally above one (ranging from 1.05 to 1.12), except for PTSD (OR 0.98). For reported stress leave due to acute stress reactions, a clear exposure‐response trend was apparent (OR for > 90 versus 0 days 1.14, 95% CI 1.02–1.26, p‐trend 4 × 10^−4^), whereas for stress‐induced exhaustion disorder and unspecified reactions to severe stress, the highest risk was seen for 1–30 versus 0 days reported (adjusted OR 1.21, 95% CI 1.02–1.44, p‐trend 0.003, and 1.17, 95% CI 1.05–1.30, p‐trend 0.004, respectively). The shift in reporting between specific diagnosis codes for stress within F43 during the period 2005–2010 (underlying our decision to exclude exposures prior to 2010 in these analyses) is illustrated in Figure S2.

**TABLE 2:** Overall risk of cancer by specific types of prior stress leave.

Stress leave by specific types of F43 diagnosis code^a^	Case/control	Adjusted OR^b^ (95% CI)	P trend^c^
Acute stress reaction (F43.0)			
No reported leave	284,887/1,316,632	1 (Reference)	
Any reported leave	1464/5848	1.11 (1.05–1.18)	
1–30 days	467/1916	1.09 (0.98–1.20)	4.0 × 10^−4^
31–90 days	565/2243	1.12 (1.02–1.22)	
> 90	432/1689	1.14 (1.02–1.26)	
Post‐traumatic stress disorder (F43.1)			
No reported leave	281,759/1,283,024	1 (Reference)	
Any reported leave	159/774	0.98 (0.83–1.16)	
1–30 days	16/62	1.17 (0.67–2.04)	0.843
31–90 days	28/161	0.80 (0.53–1.19)	
> 90	115/551	1.01 (0.83–1.24)	
Adjustment disorder (F43.2)			
No reported leave	281,896/1,284,074	1 (Reference)	
Any reported leave	167/642	1.12 (0.95–1.33)	
1–30 days	32/159	0.87 (0.59–1.27)	0.060
31–90 days	60/234	1.08 (0.81–1.44)	
> 90	75/249	1.32 (1.02–1.72)	
Stress‐induced exhaustion syndrome (F43.8A)			
No reported leave	284,222/1,312,036	1 (Reference)	
Any reported leave	1507/6001	1.10 (1.04–1.17)	
1–30 days	165/599	1.21 (1.02–1.44)	0.003
31–90 days	391/1549	1.12 (1.00–1.25)	
> 90	951/3853	1.08 (1.01–1.16)	
Specified reactions to severe stress other than exhaustion syndrome (F43.8 W)			
No reported leave	281,668/1,281,696	1 (Reference)	
Any reported leave	31/127	1.04 (0.70–1.54)	
1–30 days	8/23	1.54 (0.69–3.45)	0.844
31–90 days	11/42	1.07 (0.55–2.09)	
> 90	12/62	0.83 (0.45–1.55)	
Unspecified reactions to severe stress (F43.9)			
No reported leave	283,910/1,307,642	1 (Reference)	
Any reported leave	1458/5859	1.10 (1.04–1.16)	
1–30 days	434/1652	1.17 (1.05–1.30)	0.004
31–90 days	499/2171	1.02 (0.92–1.12)	
> 90	525/2036	1.13 (1.03–1.25)	

^a^ Stress leave is specified by the subclassification of ICD10 code F43 (reaction to severe stress and adjustment disorders). In Sweden, the first 2 weeks of sick leave are compensated by the employer and are therefore not included in the national registry data used here. Sick leave during the 1 year prior to cancer diagnosis was excluded to account for potential reverse causation.

^b^ Conditional logistic regression, conditioned on matching factors (sex, age at diagnosis and county of residence) and adjusting for socioeconomic factors (level of education, country of birth, marital status and family disposable income), was used to calculate odds ratio (OR) with 95% confidence interval (CI).

^c^ P‐trend was calculated by including the categories of stress‐leave duration as an ordinal continuous variable coded 0–3 for no stress leave to > 90 days in the multivariable analysis.

**FIGURE 3:**
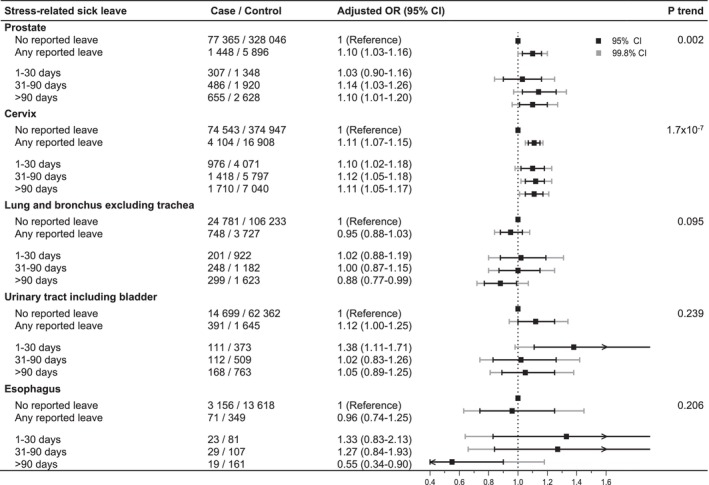
Selected associations between stress leave and subsequent risk of specific types of cancer. Stress leave is defined as registered sick leave due to psychological stress with diagnosis codes (ICD‐10, International Classification of Diseases, 10th Revision) of F43, reaction to severe stress and adjustment disorders. In Sweden, the first 2 weeks of sick leave are compensated by the employer and are therefore not included in the national registry data used here. Stress leave during the 1 year prior to cancer diagnosis was excluded to account for potential reverse causation. Conditional logistic regression, conditioned on matching factors (sex, age at diagnosis and county of residence) and adjusting for socioeconomic factors (level of education, country of birth, marital status and family disposable income), was used to calculate odds ratio (OR) with 95% confidence interval (CI). P‐trend was calculated by including the categories of stress‐leave duration as an ordinal continuous variable coded 0–3 for no stress leave to > 90 days in the multivariable analysis; the 99.8% CI was based on a Bonferroni‐corrected significance level of 0.002 (alpha value divided by the number of tumor sites = 0.05/24) to control for multiple testing. No suggestive associations were observed for the other cancer types assessed, including common diagnoses such as breast and colorectal cancer, and those results were, therefore, tabulated in Table 3 and Table S3.

Odds ratios were calculated for stress leave in relation to the risk of 24 specific types of cancer. Main findings are highlighted in Figure 3, and complete results are shown in Table 3 (any versus no reported stress leave) and Table S3 (categories of stress‐leave duration). Reported stress leave was associated with an increased risk of prostate cancer, which was apparent at durations of more than 30 days (adjusted OR for 31–90 versus 0 days 1.14, 95% CI 1.03–1.26, OR for > 90 versus 0 days 1.10, 95% CI 1.01–1.20, p‐trend 0.002). After applying the Bonferroni‐corrected significance threshold of *p* = 0.002, only the overall association for prostate cancer (any versus no stress leave) and p‐trend remained borderline significant. Reported stress leave was also positively associated with the risk of cervical cancer (adjusted OR for any versus none r 1.11, 95% CI 1.07–1.15) with similar magnitudes of association across categories of stress‐leave duration and all but the category for 1–30 days retaining statistical significance after Bonferroni correction. In further post hoc analyses, the observed association for cervical cancer was limited to carcinoma in situ (accounting for 93% of reported cervical cancer cases, adjusted OR for any versus no reported stress leave 1.12, 95% CI 1.08–1.16, consistent across exposure levels), and null for invasive cervical cancer. For lung cancer, an inverse risk relationship was found for reported stress leave > 90 days (OR 0.88, 95% CI 0.77–0.99, p‐trend = 0.095), which did not meet the Bonferroni‐corrected significance threshold. For other types of cancer, associations were generally null or had wide confidence intervals. Due to the small numbers of cases with specific types of cancers and sick leave due to specific types of psychological stress, subgroup analyses at this level of detail were not conducted.

**TABLE 3:** Overall stress leave (binary any/no) and subsequent risk of specific types of cancer.

Specific cancer types	No reported leave	Any reported leave^a^	Adjusted OR^b^ (95% CI)	Adjusted OR^b^ (99.8% CI)^c^
	Case/control	Case/control		
Prostate	77,365/328,046	1448/5896	1.10 (1.03–1.16)	1.10 (1.00–1.20)
Female breast	72,139/336,879	4197/19,851	1.00 (0.97–1.04)	1.00 (0.95–1.06)
Colon	25,666/112,092	866/3989	1.02 (0.94–1.10)	1.02 (0.90–1.15)
Rectum	16,278/72,026	506/2310	1.03 (0.93–1.14)	1.03 (0.88–1.20)
Malignant melanoma	44,107/204,407	2088/9126	1.04 (0.99–1.09)	1.04 (0.96–1.13)
Lung and bronchus (excluding trachea)	24,781/106,233	748/3727	0.95 (0.88–1.03)	0.95 (0.84–1.08)
Head and neck	10,357/46,926	305/1544	0.95 (0.84–1.08)	0.95 (0.76–1.16)
Brain	6765/31,525	212/1077	0.92 (0.79–1.07)	0.92 (0.72–1.17)
Thyroid	4767/23,012	251/1161	1.12 (0.97–1.29)	1.12 (0.89–1.40)
Esophagus	3156/13,618	71/349	0.96 (0.74–1.25)	0.96 (0.63–1.45)
Stomach‐ including cardia	5125/22,616	156/722	1.06 (0.88–1.27)	1.06 (0.80–1.41)
Pancreas	8069/35,006	263/1226	1.00 (0.87–1.15)	1.00 (0.81–1.25)
Liver	3495/15,359	83/463	0.92 (0.72–1.18)	0.92 (0.63–1.35)
Biliary tract	3598/15,699	110/601	0.85 (0.69–1.05)	0.85 (0.61–1.18)
Kidney except renal pelvis	8199/36,705	262/1191	1.06 (0.93–1.22)	1.06 (0.85–1.33)
Urinary tract including bladder	14,699/62,362	391/1645	1.12 (1.00–1.25)	1.12 (0.94–1.34)
Cervix	74,543/374,947	4104/16,908	1.11 (1.07–1.15)	1.11 (1.05–1.17)
Uterus	9513/42,860	465/2324	0.95 (0.86–1.06)	0.95 (0.81–1.12)
Ovary and fallopian tube	8555/40,178	458/2110	1.03 (0.93–1.15)	1.03 (0.87–1.22)
Other non‐follicular lymphoma	5807/25,625	152/834	0.86 (0.72–1.03)	0.86 (0.65–1.14)
Multiple myeloma	3991/17,510	115/563	0.97 (0.78–1.19)	0.97 (0.70–1.34)
B‐type chronic lymphocytic leukemia/follicular lymphoma	6146/27,226	180/841	1.00 (0.85–1.18)	1.00 (0.77–1.30)
Acute myeloid leukemia/myelodysplastic syndrome (MDS)	2653/11,557	71/373	0.88 (0.67–1.14)	0.88 (0.58–1.32)
Other^d^	57,273/266,632	2129/9556	1.07 (1.02–1.12)	1.07 (0.99–1.15)

^a^ Any registered sick leave due to psychological stress with diagnosis codes (ICD‐10, International Classification of Diseases, 10th Revision) of F43, reaction to severe stress, and adjustment disorders. In Sweden, the first 2 weeks of sick leave are compensated by the employer and are therefore not included in the national registry data used here. Stress leave during the 1 year prior to cancer diagnosis was excluded to account for potential reverse causation.

^b^ Conditional logistic regression, conditioned on matching factors (sex, age at diagnosis and county of residence) and adjusting for socioeconomic factors (level of education, country of birth, marital status and family disposable income), was used to calculate odds ratio (OR) with 95%confidence interval (CI).

^c^ The 99.8% CI was based on a Bonferroni‐corrected significance level of 0.002 (alpha value divided by the number of tumor sites = 0.05/24) to control for multiple testing.

^d^ Other malignancies, excluding the specified cancer types mentioned above.

To explore potential mediators of a link between stress reactions and cancer development, we grouped cancer diagnoses according to established etiological roles for tobacco smoking, alcohol consumption, obesity, gastric hyperacidity, and, in a post hoc analysis, HPV‐related cancer including and excluding cervical cancer (Table 4). Stress leave was associated with an increased risk of tobacco‐related cancer (adjusted OR for any versus no reported stress leave 1.06, 95% CI 1.03–1.09), with similar magnitudes of association across categories of stress‐leave duration. The risk estimate for stress leave and non‐cervical HPV‐related cancer was similar to that for cervical cancer but not statistically significant. None of the other etiology‐based cancer diagnosis groups demonstrated clear associations with pre‐diagnostic stress leave.

**TABLE 4:** Overall stress leave and risk of cancer grouped by etiology.

Stress leave^a^	Case/control	Adjusted OR^b^ (95% CI)	P trend^c^
Tobacco‐related cancer			
No reported leave	202,460/933,699	1 (Reference)	
Any reported leave	7974/35,250	1.06 (1.03–1.09)	
1–30 days	1918/8339	1.08 (1.02–1.13)	7.80 × 10^−5^
31–90 days	2656/11,665	1.07 (1.02–1.12)	
> 90	3400/15,246	1.05 (1.01–1.09)	
Obesity‐related cancer			
No reported leave	147,599/658,348	1 (Reference)	
Any reported leave	5971/27,783	1.02 (0.99–1.05)	
1–30 days	1417/6375	1.05 (0.99–1.11)	0.477
31–90 days	1871/8804	1.00 (0.95–1.06)	
> 90	2683/12,604	1.01 (0.97–1.05)	
Alcohol‐related cancer			
No reported leave	122,741/558,765	1 (Reference)	
Any reported leave	5765/27,197	1.01 (0.98–1.04)	
1–30 days	1338/6174	1.03 (0.97–1.09)	0.986
31–90 days	1854/8635	1.02 (0.97–1.07)	
> 90	2573/12,388	0.99 (0.95–1.03)	
Hyperacidity‐related cancer			
No reported leave	6420/28,253	1 (Reference)	
Any reported leave	186/868	1.05 (0.89–1.23)	
1–30 days	52/207	1.20 (0.88–1.63)	0.649
31–90 days	46/270	0.83 (0.60–1.14)	
> 90	88/391	1.11 (0.88–1.41)	
HPV‐related cancer			
No reported leave	79,940/399,999	1 (Reference)	
Any reported leave	4340/17,909	1.11 (1.07–1.15)	
1–30 days	1027/4285	1.10 (1.03–1.18)	6.3 × 10^−8^
31–90 days	1488/6118	1.11 (1.05–1.18)	
> 90	1825/7506	1.11 (1.05–1.17)	
Non‐cervical HPV‐related cancer			
No reported leave	5397/25,052	1 (Reference)	
Any reported leave	236/1001	1.12 (0.96–1.30)	
1–30 days	51/214	1.13 (0.83–1.54)	0.134
31–90 days	70/321	1.02 (0.79–1.33)	
> 90	115/466	1.18 (0.95–1.45)	

^a^ Stress leave is defined as registered sick leave due to psychological stress with diagnosis codes (ICD‐10, International Classification of Diseases, 10th Revision) of F43, reaction to severe stress and adjustment disorders. In Sweden, the first 2 weeks of sick leave are compensated by the employer and are therefore not included in the national registry data used here. Stress leave during the 1 year prior to cancer diagnosis was excluded to account for potential reverse causation.

^b^ Conditional logistic regression, conditioned on matching factors (sex, age at diagnosis and county of residence) and adjusting for socioeconomic factors (level of education, country of birth, marital status and family disposable income), was used to calculate the odds ratio (OR) with 95%confidence interval (CI).

^c^ P‐trend was calculated by including the categories of stress‐leave duration as an ordinal continuous variable coded 0–3 for no stress leave to > 90 days in the multivariable analysis. HPV = Human papillomavirus.

## 4 Discussion

In this Swedish national registry‐based study of stress leave (sick leave due to reaction to severe stress and adjustment disorders) reported in the Swedish Social Insurance Register in relation to subsequent cancer risk, we observed modest positive associations for cancer overall, and for prostate and cervix cancer specifically, as well as an inverse association for lung cancer that did not withstand Bonferroni correction for multiple testing. A clear exposure‐response association was apparent for duration of reported stress leave due to acute stress reactions in relation to overall cancer risk, (Figure 2) whereas longer duration of reported stress leave of any type was associated with higher overall cancer risk in men only (Figure S2), and suggestively also with higher prostate cancer risk (Figure 3).

Previous studies of stress as a potential risk factor for cancer have most often used self‐reported stress or registered stress diagnoses as exposure variables. For work stress, prospective studies have generally reported null associations with subsequent cancer risk [9, 10], whereas a large cohort study using repeated measures from the Japan Public Health Center‐based Prospective Study reported an elevated overall cancer risk in participants with long‐term high perceived stress [26]. Stress diagnoses recorded in secondary (specialist) care have been investigated using high‐quality, nationwide registry data from Denmark and Sweden [12, 27]. These diagnoses likely represent a higher severity of stress compared to self‐reported stress studied in prospective cohorts, in which severity cannot be so high as to prevent cohort participation. The Danish study reported a null association between adjustment disorder and cancer incidence [12], whereas the Swedish study found modest positive associations for stress‐related disorders, including adjustment disorder, which were attenuated in sibling‐controlled analyses [11]. For other severe types of stress, results have been mixed. For example, results for PTSD have been null in several studies [12, 13], including the present investigation (both any stress leave and long duration of stress leave), whereas parental bereavement has yielded some suggestive associations with cancer outcomes [28, 29].

Our observation of a positive association between stress leave and higher prostate cancer risk confirms and nuances previous findings using Swedish national diagnosis data for secondary (specialist) care [27]. Similarly, a Canadian case–control study reported an association between self‐reported workplace stress and higher risk of prostate cancer in patients under, but not over, 65 years at diagnosis, whereas prospective findings for stress‐related exposures and prostate cancer risk have generally been null [30]. With the probable exception of excess body fat and increased risk of advanced prostate cancer [31], there are no clear lifestyle‐related risk factors for prostate cancer. An association for psychological stress is thus unlikely to be mediated entirely by lifestyle. A direct physiological link beyond mediation by body fatness is plausible, through general pro‐carcinogenic mechanisms relating to several of the hallmarks of cancer [3, 4, 5, 6]. However, whether any such effect would be particularly relevant for prostate cancer is unclear. Given the high population prevalence of prostate cancer, including undiagnosed disease [32, 33], our findings may, speculatively, reflect a role for stress in promoting tumor progression leading to diagnosis. However, other non‐biological explanations may also be involved. For example, opportunistic prostate‐specific antigen (PSA) screening has been reported to be more common in men seeking health care for stress [26].

We also found a positive association between reported stress leave and HPV‐related cancer, with similar risk estimates for cervical cancer and non‐cervical cancer types (Table 4). Similar findings have been reported for psychological stress endpoints in other studies [28, 29] as well as in studies concerning mental illness requiring specialist care [34, 35]. A likely explanation for these findings is that patients suffering from severe stress or mental illness have lower participation in cancer screening programs [35, 36, 37] and therefore are not subjected to preventative treatments, including removal of precursor lesions of cervical cancer such as low‐grade squamous intraepithelial lesion (LSIL) and high‐grade squamous intraepithelial lesion (HSIL) [38]. This problem of higher cancer incidence associated with lower participation in preventive programs may increase even more in the future as mental illness was recently associated with lower likelihood of HPV vaccination among teenage girls [39]. Additionally, there may be biological mechanisms that partly explain the observed association, for example, related to higher prevalence of high‐risk human papillomavirus (HR‐HPV) infection [40]. This is possibly due to effects on the immune system that could increase both the likelihood of acquiring an infection and reduce the clearance rate [2, 4, 29, 41, 42, 43].

One previous Swedish register‐based study by Tian et al. [11] on psychological stress diagnoses and cancer risk reported associations between stress diagnoses and increased risk of breast, prostate, and lung cancer. In our study, we were partly able to replicate the association with prostate cancer but did not identify any associations between stress leave and breast or lung cancer risk. In addition, the results of the Tian study [10] on stress and breast cancer risk are contradictory to another prospective study on women in the UK reporting no association between the risk of breast cancer and perceived stress levels or adverse life events in the preceding 5 years [44]. These negative findings are corroborated in at least two other studies, including a 15‐year prospective study of Australian women at increased risk of familial breast cancer, which found no association between stress and breast cancer [45], and a meta‐analysis of 12 cohort studies in Europe, which found no link between work stress and the risk of lung, colorectal, breast, or prostate cancers [9]. However, a meta‐analysis of nine observational studies in Europe and North America did identify associations between work stress and the risk of lung, colorectal, and esophageal cancers [46].

The main strengths of this study were the essentially complete, nation‐wide exposure and outcome data sources, and the consequent very large sample size. Studying stress leave reported in the Swedish Social Insurance Register, rather than binary diagnosis data, enabled us to address the duration of leave and thus a range of exposure levels, including the higher end of the spectrum. Perhaps unexpectedly, the only clear exposure‐response trends observed were for stress leave in men and stress leave due to acute stress reactions, both in relation to overall cancer risk. Although other low p‐trend values were noted, they likely reflect the large sample size.

The use of stress leave data from the Swedish Social Insurance Register, rather than diagnosis data from the Swedish National Patient Register, also allowed us to capture exposures managed within the primary health care system and not solely within specialist care. However, this approach also limited the stress exposure data to the working age range, up to 65 years at baseline, limiting the generalizability to older populations. For individuals who turned 65 years of age during the follow‐up period, stress may have been underestimated, which we accounted for by matching cases and controls by age. People who were unemployed during the study period might represent another potential source of non‐differential misclassification of the exposure, possibly attenuating associations toward null or underestimating the true effect. Although physicians in Sweden assess and report the need for sick leave irrespective of whether the patient is employed, the Swedish Social Insurance Agency assesses work capacity in relation to the full labor market. Extended durations of stress leave typically require repeated medical evaluations by a physician, either for renewal or for initiating new leave in the case of multiple shorter absences. While individuals experiencing severe stress may be underrepresented in our data if another condition was the primary reason for sick leave, we are confident that our exposure data accurately capture stress‐related leave, even in the highest duration categories. Finally, the first 2 weeks of sick leave (for any reason) are reimbursed by the employer in Sweden and were thus not captured in our data. If short‐term stress leave is associated with cancer risk, then the risk estimates reported here could potentially be attenuated depending on the incidence of such short‐term stress leave, which remains unknown.

Although we were able to adjust for important covariates including socioeconomic factors, we did not account for some potential confounders, such as comorbidities that might contribute to both psychological stress and cancer development. Also, lifestyle‐related data were not available. However, although factors such as tobacco smoking, higher alcohol consumption, excess body fat, low physical activity, and poor diet might be confounders of stress‐related disorders, they can probably be expected to act primarily as mediators of an association between psychological stress and carcinogenesis. We conducted exploratory analyses of diagnosis groups based on etiological factors (tobacco use, obesity, alcohol, HPV, as well as hyperacidity), but formal mediation analyses would be of value to help discern the relative contribution of various lifestyle‐related factors to the associations between stress leave and cancer outcomes. Finally, our study would have benefited from higher resolution endpoint data, such as prostate cancer aggressiveness, histological subtypes of lung cancer, and hormone‐receptor status in breast cancer. Further investigation adding clinical data from national quality registers could provide additional insights into the putative link between psychological stress and the development of specific types of cancer.

## 5 Conclusions

In this population‐based registry study including 516,678 cancer cases, we found an association between sick leave reported in the Swedish Social Insurance Register due to reactions to severe stress and a modestly higher risk of incident cancer and of prostate and cervix cancer specifically. Regardless of whether the link is biological or reflective of lifestyle mediators or for cervical cancer, lower participation in screening, these findings suggest a potential relevance of severe stress for cancer prevention.

## Author Contributions

**Jenny Hadrevi:** data curation (equal), investigation (equal), project administration (lead), supervision (equal), validation (equal), visualization (equal), writing – original draft (lead), writing – review and editing (equal). **Sai San Moon Lu:** data curation (equal), formal analysis (lead), investigation (equal), methodology (equal), validation (equal), visualization (equal), writing – original draft (equal), writing – review and editing (equal). **Lisbeth Slunga Järvholm:** conceptualization (equal), investigation (equal), methodology (equal), validation (equal), writing – review and editing (equal). **Richard Palmqvist:** conceptualization (equal), investigation (equal), methodology (equal), validation (equal), writing – review and editing (equal). **Tommy Olsson:** conceptualization (equal), investigation (equal), methodology (equal), validation (equal), writing – review and editing (equal). **Sophia Harlid:** data curation (equal), formal analysis (equal), investigation (equal), methodology (equal), software (equal), supervision (equal), visualization (equal), writing – review and editing (equal). **Bethany Van Guelpen:** conceptualization (equal), funding acquisition (lead), investigation (equal), methodology (equal), project administration (equal), resources (lead), supervision (equal), validation (equal), writing – original draft (equal), writing – review and editing (equal).

## Ethics Statement

The study was approved by the Regional Ethical Review Board in Umeå, Sweden (Dnr: 2017‐194‐31 M and amendment 2019‐05046) and was conducted in accordance with the Declaration of Helsinki.

## Consent

The authors have nothing to report.

## Conflicts of Interest

Bethany Van Guelpen reports a lecturer honorarium from AstraZeneca AB. The other authors report no conflicts of interest.

## Supporting information


Data S1.

## Data Availability

The data that support the findings of this study are available on request from the corresponding author. The data are not publicly available due to privacy or ethical restrictions.
