# Supplementary material for: Sick Leave due to Stress and Subsequent Cancer Risk, a Swedish National Registry Study of 516,678 Cancer Cases
Source: Cancer Med. 2025 Apr 18;14(8):e70888. doi: 10.1002/cam4.70888 (PMC12006754; doi:10.1002/cam4.70888)
Supplement: Supplementary file 1 — Data S1. [file CAM4-14-e70888-s001.docx]

**Stress leave and subsequent cancer risk, a Swedish national registry study of 516 678 cancer cases**

# Supplementary figure 1

Supplementary figure 1: Number of stress-related diagnosis codes (F43 and its subclassifications) for sick leave by calendar year

In Sweden, the first two weeks of sick leave are compensated by the employer and are therefore not included in the national registry data used here.

# Supplementary figure 2


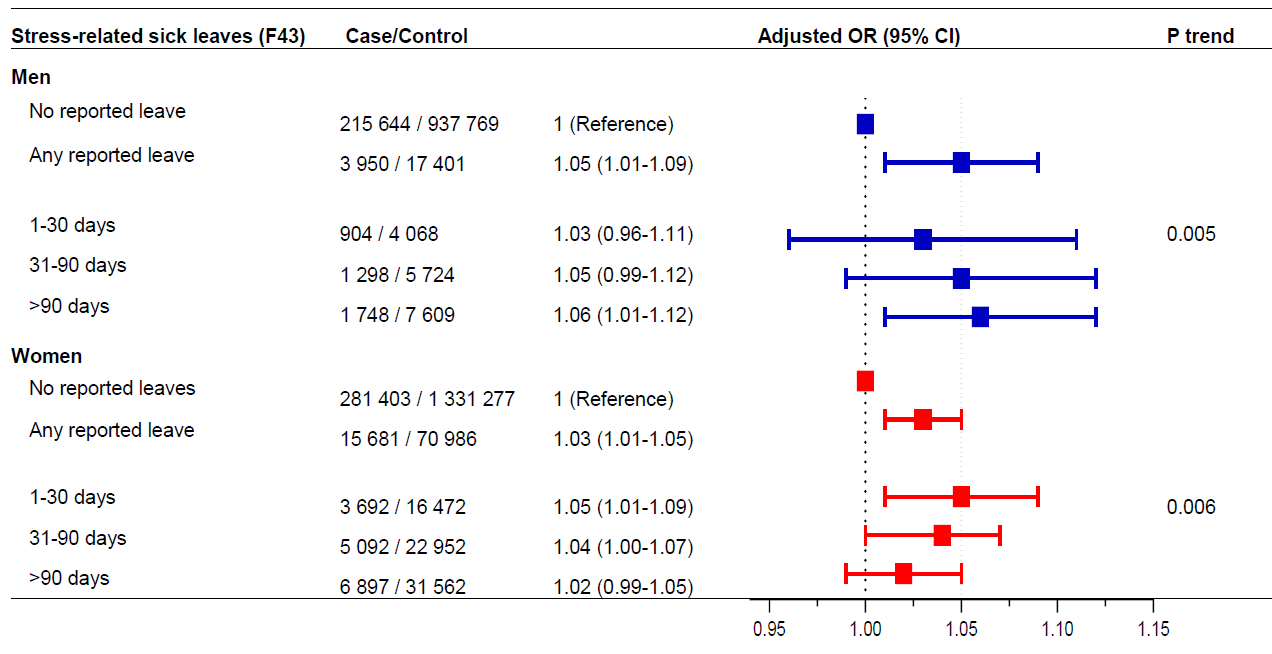


Supplementary figure 2: Stress-related sick leave in relation to overall cancer risk results by sex

Stress-related sick leave is defined as registered sick leave due to psychological stress with diagnosis codes (ICD-10, International Classification of Diseases,10^th^ Revision) of F43, reaction to severe stress and adjustment disorders. In Sweden, the first two weeks of sick leave are compensated by the employer and are therefore not included in the national registry data used here. Sick leave during the 1 year prior to cancer diagnosis was excluded to account for potential reverse causation. Conditional logistic regression, conditioned on matching factors (sex, age at diagnosis and county of residence) and adjusting for socioeconomic factors (level of education, country of birth, marital status and family disposable income), was used to calculate odds ratio (OR) with 95%confidence interval (CI). P-trend was calculated by including the categories of stress-leave duration as an ordinal continuous variable coded 0-3 for no stress leave to >90 days in the multivariable analysis.

# Supplementary table 1

Supplementary table1: Cancer diagnoses code list

| **Cancer groups** | **ICDO-10 codes^a^** | **N= 516 678** |
| --- | --- | --- |
| Prostate | C61 | 78 813 |
| Female breast | C50 | 76 336 |
| Colon | C18.0, C18.2-C18.9 | 26 532 |
| Rectum | C19, C20 | 16 784 |
| Malignant melanoma | C43 | 46 195 |
| Lung and bronchus (excluding trachea) | C34 | 25 529 |
| Head and neck | C01-C14, C30.0, C31, C32.0, C32.1, C32.2, C32.8, C32.9 | 10 662 |
| Brain | C71 | 6 977 |
| Thyroid | C73 | 5 018 |
| Esophagus | C15 | 3 227 |
| Stomach, including cardia | C16 | 5 281 |
| Pancreas | C25 | 8 332 |
| Liver | C22.0, C22.9 | 3 578 |
| Biliary tract | C22.1, C23.9, C24.0 to C24.9 | 3 708 |
| Kidney except renal pelvis | C64 | 8 461 |
| Urinary tract including bladder | C65.9, C66.9, C67, C68 | 15 090 |
| Cervix | C53 | 78 647 |
| *Carcinoma in situ (cervix)^b^* |  | 73 519 |
| *Invasive (cervix)^b^* |  | 5 128 |
| Uterus | C54 | 9 978 |
| Ovary and fallopian tube | C56, C57.0 | 9 013 |
| Other non-follicular lymphoma | C83.8 | 5 959 |
| Multiple Myeloma | C90.0 | 4 106 |
| B-type chronic lymphocytic leukemia/Follicular lymphoma | C91.1, C82 | 6 326 |
| Acute myeloid leukemia/Myelodysplastic syndrome (MDS) | C92.0, D46 | 2 724 |
| Other^c^ | Other malignancy codes | 59 402 |

^a^International Classification of Diseases for Oncology, 10^th^ Revision

^b^Carcinoma in situ and invasive cervical cancer are classified based on histopathological diagnosis codes, as defined in the “Coding in Cancer Register 2023” by the Swedish National Board of Health and Welfare

^c^Other malignancy codes, excluding the specified cancer codes mentioned above

# Supplementary table 2

Supplementary table2: Etiology-based cancer diagnosis groups

| **Etiology-based cancer groups** | **ICDO-10** |
| --- | --- |
| **Tobacoo-related cancer (n=339 992)** |  |
| Lip/Oral cavity/Pharynx | C00-C14 |
| Liver/Intrahepatic bile ducts | C22.0, C22.9, C24.0 |
| Larynx/Trachea/Bronchus/Lung | C32-C34 |
| Cervix | C53 |
| Colorectum | C18.0, C18.2-C18.9, C19, C20 |
| Kidney | C64 |
| Esophagus | C15 |
| Pancreas | C25 |
| Stomach | C16 |
| Urinary bladder | C67 |
| Acute and chronic myeloid leukemia | C91-95 and D46, excluding C91.4 |
| **Alcohol-related cancer (n=216 386)** |  |
| Oral cavity/Pharynx | C04, C14 |
| Layrnx | C32 excluding C32.3 |
| Esophagus | C15 |
| Liver | C22.0, C22.9 |
| Colorectum | C18.0, C18.2-C18.9, C19, C20 |
| Female breast | C50 |
| **Obesity-related cancer (n=278 809)** |  |
| Esophagus | C15 |
| Stomach | C16 |
| Colorectum | C18.0, C18.2-C18.9, C19, C20 |
| Liver | C22.0, C22.9 |
| Gallbladder | C23-C24 |
| Pancreas | C25 |
| Female breast (post-menopausal, approximated as breast cancers diagnosed after the age of 55 years) | C50 |
| Endometrium | C54 |
| Ovary | C56 |
| Kidney | C64 |
| Meningioma | C70.0 |
| Thyroid | C73 |
| Multiple Myeloma | C90.0 |
| **Hyper-acidity related cancer (n=13 683)** |  |
| Lower esophagus, cardia | C15.5, C16.0 |
| Stomach | C16 |
| Duodenum | C17.0 |
| **HPV-related cancer (n=84 280)** |  |
| Cervix | C53 |
| Anus | C21 |
| Vagina | C52 |
| Oropharynx | C01.9,C02.4,C02.8,C05.1,C05.2,C05.9,C09,C10.0,C10.2,  C10.8,C10.9,C14.2 |
| Vulva | C51 |
| Penis | C60 |

HPV=Human papillomavirus

# Supplementary table 3

Supplementary table 3: Overall stress-related sick leave and risk for specific types of cancer

| **Stress-related sick leave** | **Case / Control** | **Adjusted OR (95% CI)** | **Adjusted OR (99.8% CI)** | **P trend** |
| --- | --- | --- | --- | --- |
| Prostate |  |  |  |  |
| No reported leave | 77 365 / 328 046 | 1 (Reference) | 1 (Reference) | 0.002 |
| 1-30 days | 307 / 1 348 | 1.03 (0.90-1.16) | 1.03 (0.84-1.25) |  |
| 31-90 days | 486 / 1 920 | 1.14 (1.03-1.26) | 1.14 (0.97-1.33) |  |
| >90 days | 655 / 2 628 | 1.10 (1.01-1.20) | 1.10 (0.96-1.27) |  |
| Female breast |  |  |  |  |
| No reported leave | 72 139 / 336 879 | 1 (Reference) | 1 (Reference) | 0.858 |
| 1-30 days | 973 / 4 459 | 1.04 (0.97-1.11) | 1.04 (0.93-1.16) |  |
| 31-90 days | 1 331 / 6 304 | 1.00 (0.94-1.06) | 1.00 (0.91-1.10) |  |
| >90 days | 1 893 / 9 088 | 0.99 (0.94-1.04) | 0.99 (0.91-1.07) |  |
| Colon |  |  |  |  |
| No reported leave | 25 666 / 112 092 | 1 (Reference) | 1 (Reference) |  |
| 1-30 days | 205 / 927 | 1.04 (0.89-1.21) | 1.04 (0.82-1.32) | 0.798 |
| 31-90 days | 277 / 1 251 | 1.03 (0.90-1.18) | 1.03 (0.84-1.27) |  |
| >90 days | 384 / 1 811 | 1.00 (0.89-1.12) | 1.00 (0.84-1.19) |  |
| Rectum |  |  |  |  |
| No reported leave | 16 278 / 72 026 | 1 (Reference) | 1 (Reference) | 0.508 |
| 1-30 days | 109 / 532 | 0.96 (0.78-1.19) | 0.96 (0.69-1.34) |  |
| 31-90 days | 169 / 740 | 1.07 (0.91-1.27) | 1.07 (0.82-1.40) |  |
| >90 days | 228 / 1 038 | 1.03 (0.89-1.19) | 1.03 (0.82-1.30) |  |
| Malignant melanoma |  |  |  |  |
| No reported leave | 44 107 / 204 407 | 1 (Reference) | 1 (Reference) | 0.087 |
| 1-30 days | 468 / 2 153 | 1.00 (0.90-1.10) | 1.00 (0.85-1.17) |  |
| 31-90 days | 684 / 2 928 | 1.06 (0.97-1.16) | 1.06 (0.93-1.21) |  |
| >90 days | 936 / 4 045 | 1.05 (0.98-1.13) | 1.05 (0.93-1.18) |  |
| Lung and bronchus (excluding trachea) |  |  |  |  |
| No reported leave | 24 781 / 106 233 | 1 (Reference) | 1 (Reference) | 0.095 |
| 1-30 days | 201 / 922 | 1.02 (0.88-1.19) | 1.02 (0.80-1.31) |  |
| 31-90 days | 248 / 1 182 | 1.00 (0.87-1.15) | 1.00 (0.80-1.25) |  |
| >90 days | 299 / 1 623 | 0.88 (0.77-0.99) | 0.88 (0.72-1.07) |  |
| Head and neck |  |  |  |  |
| No reported leave | 10 357 / 46 926 | 1 (Reference) | 1 (Reference) | 0.467 |
| 1-30 days | 62 / 353 | 0.85 (0.65-1.11) | 0.85 (0.55-1.30) |  |
| 31-90 days | 109 / 474 | 1.10 (0.89-1.36) | 1.10 (0.79-1.53) |  |
| >90 days | 134 / 717 | 0.90 (0.75-1.09) | 0.90 (0.67-1.21) |  |
| Brain |  |  |  |  |
| No reported leave | 6 765 / 31 525 | 1 (Reference) | 1 (Reference) | 0.579 |
| 1-30 days | 46 / 266 | 0.81 (0.59-1.11) | 0.81 (0.49-1.33) |  |
| 31-90 days | 66 / 366 | 0.84 (0.65-1.10) | 0.84 (0.56-1.28) |  |
| >90 days | 100 / 445 | 1.04 (0.83-1.30) | 1.04 (0.73-1.48) |  |

Supplementary table 3: Overall stress-related sick leave and risk for specific types of cancer (continued)

| **Stress-related sick leave** | **Case / Control** | **Adjusted OR (95% CI)** | **Adjusted OR (99.8% CI)** | **P trend** |
| --- | --- | --- | --- | --- |
| Thyroid |  |  |  |  |
| No reported leave | 4 767 / 23 012 | 1 (Reference) | 1 (Reference) | 0.205 |
| 1-30 days | 54 / 235 | 1.19 (0.88-1.61) | 1.19 (0.74-1.92) |  |
| 31-90 days | 85 / 388 | 1.13 (0.89-1.44) | 1.13 (0.78-1.65) |  |
| >90 days | 112 / 538 | 1.08 (0.88-1.33) | 1.08 (0.78-1.50) |  |
| Esophagus |  |  |  |  |
| No reported leave | 3 156 / 13 618 | 1 (Reference) | 1 (Reference) | 0.206 |
| 1-30 days | 23 / 81 | 1.33 (0.83-2.13) | 1.33 (0.64-2.80) |  |
| 31-90 days | 29 / 107 | 1.27 (0.84-1.93) | 1.27 (0.66-2.45) |  |
| >90 days | 19 / 161 | 0.55 (0.34-0.90) | 0.55 (0.26-1.18) |  |
| Stomach- including cardia |  |  |  |  |
| No reported leave | 5 125 / 22 616 | 1 (Reference) | 1 (Reference) | 0.480 |
| 1-30 days | 43 / 170 | 1.23 (0.88-1.73) | 1.23 (0.72-2.10) |  |
| 31-90 days | 34 / 228 | 0.72 (0.50-1.04) | 0.72 (0.40-1.28) |  |
| >90 days | 79 / 324 | 1.21 (0.94-1.55) | 1.21 (0.81-1.80) |  |
| Pancreas |  |  |  |  |
| No reported leave | 8 069 / 35 006 | 1 (Reference) | 1 (Reference) | 0.904 |
| 1-30 days | 60 / 266 | 1.05 (0.80-1.40) | 1.05 (0.68-1.65) |  |
| 31-90 days | 76 / 399 | 0.91 (0.71-1.16) | 0.91 (0.61-1.34) |  |
| >90 days | 127 / 561 | 1.05 (0.86-1.28) | 1.05 (0.77-1.43) |  |
| Liver |  |  |  |  |
| No reported leave | 3 495 / 15 359 | 1 (Reference) | 1 (Reference) | 0.576 |
| 1-30 days | 19 / 113 | 0.83 (0.50-1.36) | 0.83 (0.38-1.82) |  |
| 31-90 days | 31 / 159 | 1.00 (0.68-1.49) | 1.00 (0.54-1.87) |  |
| >90 days | 33 / 191 | 0.90 (0.62-1.33) | 0.90 (0.49-1.66) |  |
| Biliary tract |  |  |  |  |
| No reported leave | 3 598 / 15 699 | 1 (Reference) | 1 (Reference) | 0.221 |
| 1-30 days | 27 / 161 | 0.77 (0.51-1.17) | 0.77 (0.40-1.48) |  |
| 31-90 days | 33 / 177 | 0.85 (0.59-1.24) | 0.85 (0.47-1.54) |  |
| >90 days | 50 / 263 | 0.89 (0.66-1.22) | 0.89 (0.55-1.45) |  |
| Kidney except renal pelvis |  |  |  |  |
| No reported leave | 8 199 / 36 705 | 1 (Reference) | 1 (Reference) | 0.395 |
| 1-30 days | 54 / 249 | 1.03 (0.77-1.39) | 1.03 (0.65-1.65) |  |
| 31-90 days | 91 / 406 | 1.09 (0.87-1.37) | 1.09 (0.76-1.57) |  |
| >90 days | 117 / 536 | 1.06 (0.86-1.30) | 1.06 (0.77-1.46) |  |
| Urinary tract including bladder |  |  |  |  |
| No reported leave | 14 699 / 62 362 | 1 (Reference) | 1 (Reference) | 0.239 |
| 1-30 days | 111 / 373 | 1.38 (1.11-1.71) | 1.38 (0.98-1.93) |  |
| 31-90 days | 112 / 509 | 1.02 (0.83-1.26) | 1.02 (0.74-1.42) |  |
| >90 days | 168 / 763 | 1.05 (0.89-1.25) | 1.05 (0.81-1.38) |  |

Supplementary table 3: Overall stress-related sick leave and risk for specific types of cancer (continued)

| **Stress-related sick leave** | **Case / Control** | **Adjusted OR (95% CI)** | **Adjusted OR (99.8% CI)** | **P trend** |
| --- | --- | --- | --- | --- |
| Cervix |  |  |  |  |
| No reported leave | 74 543 / 374 947 | 1 (Reference) | 1 (Reference) | 1.7x10^-7^ |
| 1-30 days | 976 / 4 071 | 1.10 (1.02-1.18) | 1.10 (0.98-1.23) |  |
| 31-90 days | 1 418 / 5 797 | 1.12 (1.05-1.18) | 1.12 (1.02-1.23) |  |
| >90 days | 1 710 / 7 040 | 1.11 (1.05-1.17) | 1.11 (1.01-1.21) |  |
| *Carcinoma in situ (cervix)* |  |  |  |  |
| *No reported leave* | *69 712 / 351 283* | *1 (Reference)* | *1 (Reference)* | *9.2x10^-8^* |
| *1-30 days* | *917 / 3 755* | *1.12 (1.04-1.20)* | *1.12 (1.00-1.26)* |  |
| *31-90 days* | *1 309 / 5 340* | *1.12 (1.05-1.19)* | *1.12 (1.01-1.23)* |  |
| *>90 days* | *1 581 / 6 436* | *1.11 (1.05-1.18)* | *1.11 (1.02-1.22)* |  |
| *Invasive (cervix)* |  |  |  |  |
| *No reported leave* | *4 831 / 23 664* | *1 (Reference)* | *1 (Reference)* | *0.628* |
| *1-30 days* | *59 / 316* | *0.88 (0.66-1.16)* | *0.88 (0.56-1.37)* |  |
| *31-90 days* | *109 / 457* | *1.12 (0.90-1.39)* | *1.12 (0.80-1.57)* |  |
| *>90 days* | *129 / 604* | *1.02 (0.84-1.24)* | *1.02 (0.75-1.39)* |  |
| Uterus |  |  |  |  |
| No reported leave | 9 513 / 42 860 | 1 (Reference) | 1 (Reference) | 0.237 |
| 1-30 days | 118 / 577 | 0.96 (0.79-1.18) | 0.96 (0.70-1.32) |  |
| 31-90 days | 153 / 697 | 1.06 (0.88-1.26) | 1.06 (0.80-1.4) |  |
| >90 days | 194 / 1 050 | 0.88 (0.75-1.03) | 0.88 (0.69-1.12) |  |
| Ovary and fallopian tube |  |  |  |  |
| No reported leave | 8 555 / 40 178 | 1 (Reference) | 1 (Reference) | 0.896 |
| 1-30 days | 119 / 473 | 1.20 (0.98-1.46) | 1.20 (0.87-1.65) |  |
| 31-90 days | 140 / 693 | 0.97 (0.80-1.16) | 0.97 (0.72-1.29) |  |
| >90 days | 199 / 944 | 1.00 (0.85-1.17) | 1.00 (0.78-1.28) |  |
| Other non-follicular lymphoma |  |  |  |  |
| No reported leave | 5 807 / 25 625 | 1 (Reference) | 1 (Reference) | 0.110 |
| 1-30 days | 34 / 195 | 0.82 (0.57-1.19) | 0.82 (0.46-1.47) |  |
| 31-90 days | 53 / 262 | 0.95 (0.70-1.28) | 0.95 (0.59-1.52) |  |
| >90 days | 65 / 377 | 0.82 (0.63-1.07) | 0.82 (0.54-1.25) |  |
| Multiple Myeloma |  |  |  |  |
| No reported leave | 3 991 / 17 510 | 1 (Reference) | 1 (Reference) | 0.839 |
| 1-30 days | 24 / 137 | 0.81 (0.52-1.26) | 0.81 (0.41-1.62) |  |
| 31-90 days | 40 / 167 | 1.15 (0.81-1.63) | 1.15 (0.66-2.00) |  |
| >90 days | 51 / 259 | 0.93 (0.69-1.27) | 0.93 (0.58-1.51) |  |
| B-type chronic lymphocytic leukemia/Follicular lymphoma | | |  |  |
| No reported leave | 6 146 / 27 226 | 1 (Reference) | 1 (Reference) | 0.863 |
| 1-30 days | 41 / 207 | 0.93 (0.66-1.30) | 0.93 (0.54-1.57) |  |
| 31-90 days | 61 / 279 | 1.02 (0.77-1.35) | 1.02 (0.66-1.59) |  |
| >90 days | 78 / 355 | 1.03 (0.80-1.32) | 1.03 (0.69-1.52) |  |
| Acute myeloid leukemia/Myelodysplastic syndrome (MDS) | | |  |  |
| No reported leave | 2 653 / 11 557 | 1 (Reference) | 1 (Reference) | 0.323 |
| 1-30 days | 20 / 82 | 1.12 (0.68-1.83) | 1.12 (0.52-2.43) |  |
| 31-90 days | 17 / 131 | 0.60 (0.36-1.00) | 0.60 (0.27-1.34) |  |
| >90 days | 34 / 160 | 0.97 (0.67-1.41) | 0.97 (0.54-1.75) |  |
| Other |  |  |  |  |
| No reported leave | 57 273 / 266 632 | 1 (Reference) | 1 (Reference) | 0.007 |
| 1-30 days | 502 / 2 190 | 1.10 (1.00-1.21) | 1.10 (0.94-1.28) |  |
| 31-90 days | 647 / 3 112 | 1.00 (0.92-1.09) | 1.00 (0.87-1.14) |  |
| >90 days | 980 / 4 254 | 1.11 (1.03-1.19) | 1.11 (0.99-1.24) |  |
